# Comparison of standard-dose and reduced-dose treatment of metastatic prostate cancer with enzalutamide, apalutamide or darolutamide: a rapid review

**DOI:** 10.1136/bmjonc-2023-000198

**Published:** 2024-02-29

**Authors:** Hannah Louise Bromley, Mohini Varughese, Duncan C Gilbert, Peter Hoskin, Ian F Tannock, Kimberley Reeves, Ananya Choudhury

**Affiliations:** 1 Division of Cancer Sciences, The University of Manchester, Manchester, UK; 2 The Christie NHS Foundation Trust, Manchester, UK; 3 Royal Devon University Healthcare NHS Foundation Trust, Exeter, UK; 4 MRC Clinical Trials Unit at UCL, UCL, London, UK; 5 Mount Vernon Cancer Centre, Northwood, UK; 6 Princess Margaret Cancer Centre, Toronto, Ontario, Canada; 7 Clinical Oncology, The Christie NHS Foundation Trust, Manchester, UK

**Keywords:** Prostate cancer

## Abstract

**Objective:**

To review the efficacy and safety of low-dose versus standard-dose enzalutamide, apalutamide or darolutamide treatment for metastatic prostate cancer.

**Methods and analysis:**

Keyword searches in MEDLINE and EMBASE up to 1 June 2023, with forward and backward citation searches of potentially relevant studies. Studies were included if primary outcome data were reported for patients with metastatic prostate cancer who had received reduced doses of enzalutamide, apalutamide or darolutamide. Searches were limited to original full-text and English-language studies. Key outcomes included overall survival (OS), progression-free survival (PFS), prostate-specific antigen response and treatment-related adverse events. The review was performed in accordance with Cochrane Rapid Reviews Methods Group guidelines.

**Results:**

Ten studies were identified that met the eligibility criteria: five phase I studies, two post-hoc analyses of phase III trials and three retrospective analyses. No consistent association between OS, PFS and drug dose was identified. Fewer severe treatment-related adverse events were observed at lower drug doses.

**Conclusion:**

This review provides evidence that enzalutamide, apalutamide or darolutamide could be given at a lower than the standard recommended dose without loss of antitumour activity. A prospective near-equivalence randomised trial should be undertaken to compare registered and lower doses of these agents.

**PROSPERO registration number:**

CRD42023440371.

**WHAT IS ALREADY KNOWN ON THIS TOPIC**

**WHAT THIS STUDY ADDS**

- Enzalutamide, apalutamide or darolutamide could be given at a lower dose without likely loss of antitumour activity.
- There was no consistent association between overall survival, progression-free survival and drug dose in included studies.
- Fewer severe treatment-related adverse events were observed at lower drug doses.

**HOW THIS STUDY MIGHT AFFECT RESEARCH, PRACTICE OR POLICY**

- A prospective randomised trial of reduced and standard doses is needed to validate the efficacy of lower-dose regimens.

## Introduction

 Enzalutamide, apalutamide and darolutamide are androgen receptor (AR) inhibitors which improve overall survival (OS) of men with metastatic prostate cancer, when added to standard androgen deprivation therapy (ADT) in randomised controlled trials (RCTs).^1^,^6^ The recommended doses of enzalutamide (160 mg/day), apalutamide (240 mg/day) and darolutamide (1200 mg/day) were established in phase I trials.^3 7 8^ Enzalutamide improves OS in men with castrate-resistant prostate cancer (CRPC) before and after docetaxel chemotherapy, and in metastatic hormone-sensitive prostate cancer (MHSPC).^1 9^ Apalutamide and darolutamide demonstrated increased OS in men with MHSPC.^4 5^ All three drugs also improve metastasis-free survival in men with non-metastatic CRPC and a rapidly rising prostate-specific antigen (PSA).^6 10 11^

Although effective and relatively well tolerated compared with other anticancer drugs, each of the ’utamides has associated toxicities and in practice, lower doses may be prescribed in elderly patients or those with comorbidity or poor performance status.^12^ Enzalutamide increases fatigue,^3 13^ which may improve with dose reduction.^14^ Apalutamide can cause a skin rash or other dermatological side-effects, including pruritus, in about one in four men,^2 15^ requiring either treatment interruption or dose reduction. There are claims that darolutamide is less toxic, and that it does not cross the blood–brain barrier, although there is no robust clinical evidence to support this claim.^16^ All three drugs increase the risk of cardiovascular events and hypertension.^17^

This rapid review summarises outcomes of men with metastatic prostate cancer, treated with enzalutamide, apalutamide or darolutamide at a lower than the recommended dose.

## Methods

### Study design

This study adopted guidelines from the Cochrane Rapid Reviews Methods Group.^18^ Rapid reviews enable an efficient and pragmatic approach to evidence synthesis, in which key components of a systematic review are simplified,^19^ allowing a clinical question to be addressed rapidly. The study protocol was registered on PROSPERO (ID number: CRD42023440371), to promote reproducibility and methodological transparency.^20^

### Search strategy

Keyword searches together with Boolean operators (OR±AND) and truncation (*) were conducted to identify English-language peer-reviewed literature, related to standard and lower-dose enzalutamide, apalutamide and darolutamide treatment of men with metastatic prostate cancer.

MEDLINE and EMBASE electronic databases were searched for studies published up to 1 April 2023 and updated on 1 June 2023. Studies were limited to original full-text and English-language records. Keyword searches included “prostat* adj3 (cancer* or tumo* or neoplas* or carcinom* or malign*” OR “prostate cancer/” AND “Androgen Antagonists/” OR (enzalutamide or apalutamide or darolutamide” AND “dos* OR Dose-Response Relationship, Drug/ OR Drug dose regimen/”.

### Study selection

All retrieved studies were collated and managed via EndNote referencing software.^21^ A two-stage process was used to identify relevant studies for inclusion in the final review.^18^ First, the title and abstracts were screened against the eligibility criteria, and then the full text of possible relevant studies was assessed for relevance. The reference lists of potentially relevant studies were also searched. Studies reporting primary outcomes which met the eligibility criteria were included in the final review. A second reviewer checked a sample of studies to investigate any selection bias, and disagreements were resolved through discussion.

### Eligibility criteria

Studies were included if they met the following inclusion criteria:

- Population: adult men (aged >18 years) with a diagnosis of metastatic prostate cancer.
- Intervention: reduced doses of enzalutamide, apalutamide of darolutamide±standard ADT.
- Comparator: recommended doses of enzalutamide, apalutamide or darolutamide.
- Outcomes: any primary data reported on progression-free survival (PFS), OS, toxicity or adverse effects, PSA response, duration of treatment, health-related quality of life.
- Type: English language, primary/original full-text studies.

Systematic reviews, editorials, commentaries, opinion pieces and meeting abstracts were excluded.

### Data analysis

An electronic template adapted from the Cochrane Review Methods Group^18^ was used to extract data on the characteristics and outcomes from the included studies. Data on study methods, participants, doses, clinical outcomes and adverse events were tabulated and the results analysed narratively. Due to the heterogeneity of study designs, patient characteristics, drug doses and outcomes, a formal meta-analysis was considered statistically inappropriate (online supplemental file 1).

A formal quality appraisal was not performed due to the scoping nature of the review and range of different study types included.

## Results

### Search results

**Figure 1:**
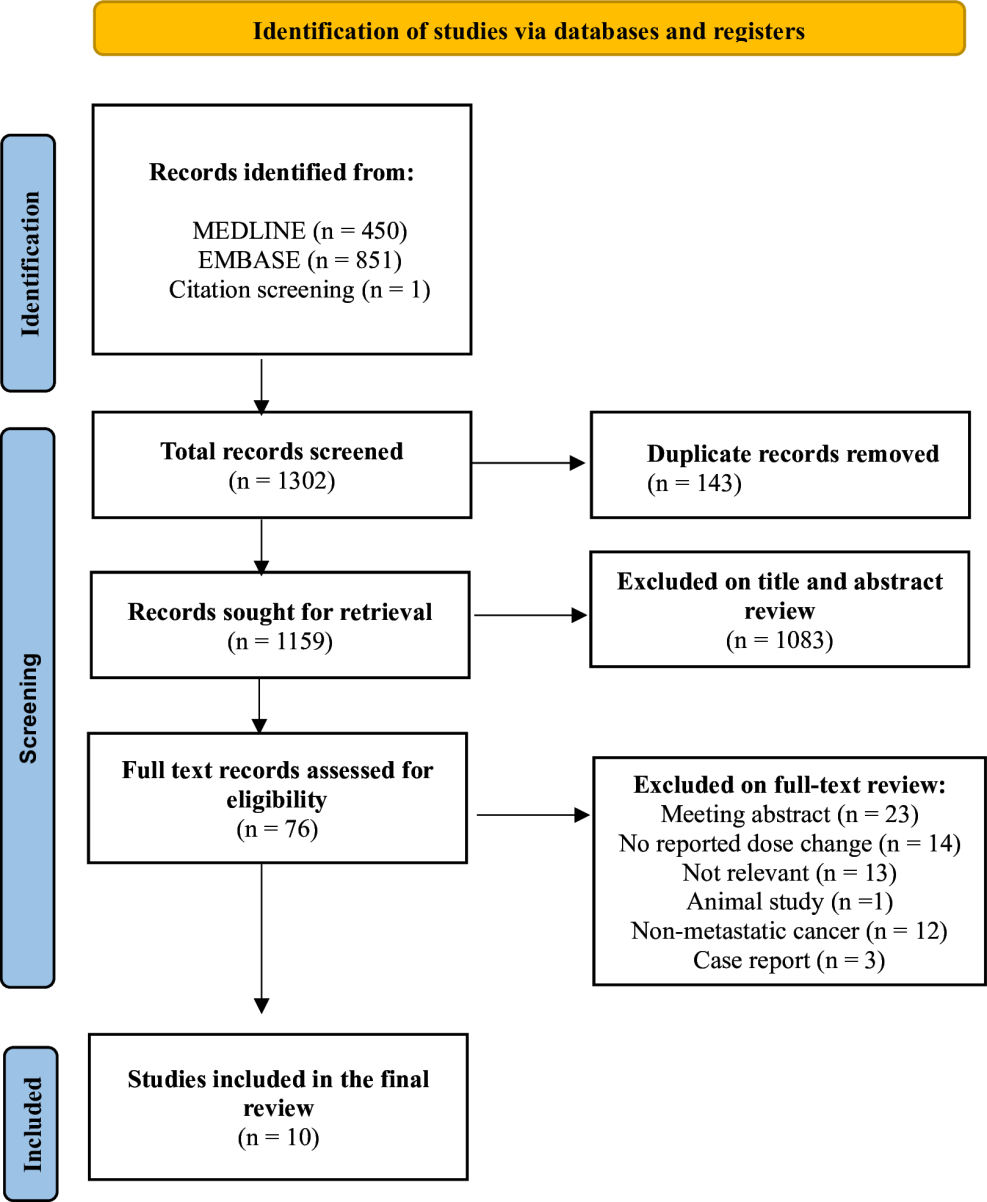
A Preferred Reporting Items for Systematic Reviews and Meta-Analyses flow chart of studies included and excluded at each stage.

A total of 1302 citations were identified in the literature search, of which 143 were removed as duplicates. Ten studies were included in the final review. Figure 1 summarises the studies included and excluded at each stage of the literature search.

### Study characteristics

A summary of the included studies, design and outcomes is provided in table 1. Evidence pertaining to clinical outcomes from reduced-dose enzalutamide, apalutamide and darolutamide is from phase I studies, post-hoc analyses of phase III trials and retrospective cohort analyses.

**Table 1:** Characteristics of included studies (n=10)

Study	Drug and dose	Population	Methods and design	Adverse events (AEs)	Key outcomes
Akaza *et al*^22^	Enzalutamide 80–240 mg	MCRPC	Multicentre open-label uncontrolled phase I/II trial in Japan; phase I (n=9), phase II (n=38) patients	Fewer AEs in phase I at low dose (22.2% vs 34.2%)	Pharmacokinetics dose proportional from 80 to 240 mg
Fizazi *et al*^8^	Darolutamide 200–1800 mg	MCRPC	Multicentre open-label uncontrolled phase I/II in Europe and the USA (n=124)	Most common AEs were fatigue (12%) or hot flushes (5%); no differences observed by dose	Phase I: no dose-limiting toxic effects, maximum tolerated dose not reached Phase II: 11 (29%) patients in 200 mg, 13 (33%) in 400 mg and 11 (33%) in 1400 mg groups achieved PSA response at 12 weeks
Fizazi *et al*^23^	Darolutamide 200–1800 mg	MCRPC	Extended follow-up of multicentre phase I/II trial in CYP17 inhibitor-naïve patients (n=77)	The most common AEs: fatigue, diarrhoea, anorexia, hot flushes	PSA progression, HR 0.47 (95% CI 0.12 to 1.82; p=0.2743) for 1400 mg group and 1.32 (95% CI 0.48 to 3.62; p=0.596) for the 400 mg group compared with the 200 mg/day group
Freedland *et al*^26^	Enzalutamide reduced dose (<80%)	MCRPC	Retrospective longitudinal cohort study of US veteran health data (n=6069)	No AE reported	RDI <80% associated with higher-risk PSA progression, HR 1.258 (95% CI 1.080 to 1.464, p<0.003); no assessment of PFS or OS
Oishi *et al*^27^	Apalutamide 240 mg or reduced dose (60–180 mg/day)	MCSPC	Multicentre retrospective study (35 non-MCRPC and 72 MCRPC)	No difference in skin AE between doses (p=0.761)	No difference in skin-related AE-free survival between dose groups (p=0.33) No difference in CRPC-free survival between doses in metastatic disease (p=0.525) Drug discontinuation higher in standard (50%) versus reduced dose (16.7%) (p=0.021)
Rathkopf *et al*^7^	Apalutamide 30–480 mg/day	MCRPC	Phase I study in single centre at doses between 30 mg and 480 mg (n=30)	Most common AE was fatigue	Pharmacokinetics linear and dose proportional Dose escalation to 480 mg did not identify a maximum tolerated dose Reduced FDHT uptake at all doses, with a plateau in response at ≥120 mg
Scher *et al*^3^	Enzalutamide 30–600 mg	MCRPC	Phase I/II study undertaken in the USA (n=140)	Severe fatigue dose dependent Fatigue highest in doses ≥240 mg/day	Antitumour effects were observed at all doses Maximum tolerated dose for sustained treatment was 240 mg
T’jollyn *et al*^28^	Apalutamide 240 mg: quartiles of dose exposure	MCSPC	Retrospective analysis of phase III randomised controlled trial (n=1052), comparing quartiles of dose exposure	Skin rash and pruritus increased with increasing apalutamide exposure	No statistical association between OS and apalutamide exposure quartiles No association detected between radiographic PFS and apalutamide exposure quartiles
Vinh-Hung *et al*^24^	Enzalutamide 160 mg/day vs ≤80 mg/day	Metastatic prostate cancer	Retrospective review at a single centre in the Caribbean (n=111)	Limited data reported	No difference in OS and PFS between low dose and standard dose groups Better longevity in low dose group, RMAA 89.1 years vs 83.8 years (p=0.003)
Vinh-Hung *et al*^25^	Enzalutamide 160 mg/day vs ≤80 mg/day	Metastatic prostate cancer	Retrospective review of men aged ≥75 years at a single centre in the Caribbean (n=59)	No AEs reported in low dose group	PSA decrease ≥50% at 12 weeks in 67% with low dose vs 45% with standard dose (p=0.152) Median PFS 11.2 months in low dose group vs 11.9 months for standard dose (p=0.612)

CRPC, castrate-resistant prostate cancer; FDHT, Fluorodihydrotestosterone; MCRPC, metastatic CRPC; MCSPC, metastatic castrate-sensitive prostate cancer; OS, overall survival; PFS, progression-free survival; PSA, prostate-specific antigen; RDI, relative dose intensity; RMAA, restricted mean attained age.

### Phase I trials

Five phase I studies generated data on the clinical effectiveness of enzalutamide, apalutamide and darolutamide at lower doses.^3 7 8 22 23^

In the original phase I study of enzalutamide (formerly MDV3100),^3^ 140 men were treated with doses between 30 and 600 mg/day. Antitumour effects were observed at all doses, including decreases in serum PSA of ≥50% in over half of all patients. There was an almost linear increase in steady-state serum concentration with dose, but no trends to increased target binding or tumour response at doses from 60 mg to 360 mg/day. The registered dose is 160 mg/day. An increase in the severity of treatment-related adverse effects was associated with drug dose; grade 3–4 fatigue was associated with higher doses (≥240 mg/day). Symptoms generally resolved after dose reduction.

A similar pharmacokinetic profile was observed in a Japanese phase I/II multicentre study of enzalutamide in patients with metastatic CRPC (n=9 in phase I, n=38 in phase II).^22^ Pharmacokinetic profiles were dose proportional at doses from 80 to 240 mg/day. Fewer serious treatment-emergent adverse events were reported at lower doses of <160 mg/day. PSA reduction from baseline was observed at 160 mg/day in the phase II study, but data on antitumour activity were not reported for the lower doses in phase I.^22^

In phase I trials of apalutamide (formerly ARN-509),^7^ 30 patients received doses from 30 to 480 mg/day. Pharmacokinetics were linear and dose proportional. Antitumour activity was observed at all doses with a plateau effect ≥120 mg/day and >90% inhibition of testosterone binding to the AR in FDHT/positron emission tomography CT analysis. The registered dose is 240 mg/day.

In a phase I/II study of darolutamide (formerly ODM-201),^8^ 110 men with metastatic CRPC were randomised to receive 200, 400 or 1400 mg/day. Exposure at steady state increased in a linear, dose-related manner up to 1400 mg/day. PSA response at 12 weeks was similar at these doses (29%, 33% and 33%), although in men without prior treatment with abiraterone or similar agent, the 200 mg dose was inferior; there was no difference between 400 and 1400 mg/day. The registered dose is 1200 mg/day. No difference in adverse events was observed between groups when examined by dose or by previous treatment.^8^ Extended follow-up of 77 patients from the phase I/II trial of darolutamide was undertaken among individuals with CYP17 inhibitor-naïve CRPC.^23^ Antitumour activity was demonstrated at all doses between 200 and 1800 mg/day. However, in chemotherapy-naïve patients, there was a trend to greater PSA suppression at the higher dose of 1400 mg/day when compared with 200 mg/day (HR 0.47, 95% CI 0.12 to 1.82, p=0.27). Treatment-related toxicities were observed in 27 patients (35%). The most frequent toxicities were fatigue/asthenia (10.4%), diarrhoea (5.2%), anorexia (5.2%) and hot flushes (3.9%), but most were graded as mild and no further dose reductions were made.^23^

### Retrospective analyses

Two studies investigated retrospectively the impact of starting enzalutamide at ≤50% dose on metastatic prostate cancer outcomes.^24 25^ Of 111 patients treated in the first study, 32 received a low dose (≤80 mg/day) and 79 the standard dose (160 mg/day). Men who received low-dose enzalutamide had poorer Eastern Cooperative Oncology Group (ECOG) performance status and more comorbidities, although baseline PSA, doubling time and distribution of metastases were similar between the groups. The study found that men receiving low-dose enzalutamide had better longevity (restricted mean attained age: 89.1 years in low-dose enzalutamide vs 83.3 years in standard dose patients, p=0.025), as well as fewer adverse events.^24^ In a similar analysis of 59 patients with metastatic prostate cancer aged ≥75 years,^25^ 43 men received low-dose enzalutamide (≤80 mg/day) and 16 the standard dose (160 mg/day). PSA response (reduction of ≥50% from baseline) was observed in 18 (45%) and 10 (67%) patients receiving low and standard-dose enzalutamide at 12 weeks (p=0.15), respectively. No significant difference in OS or PSA PFS was observed between the two groups.

A retrospective, longitudinal cohort study of 6069 US veterans with metastatic CRPC explored the impact of relative dose intensity (RDI) of enzalutamide on PSA progression (defined as PSA ≥2 ng/mL and ≥25% above the nadir).^26^ In total, 924 (67.2%) men taking enzalutamide had at least one RDI <80% over at least 2 months, and this was associated with a higher risk of PSA progression (HR 1.26, 95% CI 1.09 to 1.46). No assessment of the impact of lower enzalutamide doses on PFS or OS was performed due to limited data.^26^

A post-hoc analysis of 72 patients with MHSPC explored the effect of apalutamide dose reduction on skin-related adverse events and survival, comparing two groups receiving standard dose (240 mg/day) and reduced dose (60–180 mg/day) regimens.^27^ There was no significant difference in the incidence of skin-related adverse events between the two dose groups. There were no significant differences in progression to castrate resistance among those receiving the standard or a reduced dose of apalutamide, nor between patients with and without skin-related adverse events.

### Post-hoc analysis of RCT

A post-hoc analysis of 1052 patients in the phase III randomised controlled TITAN trial examined the relationship between the pharmacokinetics of apalutamide exposure, efficacy and safety outcomes using a multivariate Cox regression model.^28^ Primary analysis of the trial found that apalutamide at a planned dose of 240 mg/day plus ADT improved survival versus placebo in patients with MHSPC,^4^ but some men received lower doses. No statistical association was detected between OS, PFS and apalutamide exposure quartiles.^28^ Incidence of skin rash and pruritus increased significantly with higher apalutamide exposure.^28^

## Discussion

This review identified peer-reviewed evidence that reported on primary outcomes related to reduced-dose enzalutamide, apalutamide and darolutamide in men with metastatic prostate cancer. The findings suggest that each drug could be given at lower than the registered dose without potential loss of antitumour activity, and with a probable decrease in toxicity due to off-target effects.

There is an unmet need for assessing the potential benefits of lower-dose anticancer treatments, particularly in frail or comorbid patients^29^ who may experience substantial toxicity when given registered doses.^30 31^ The development of anticancer drugs continues to rely on historical paradigms for cytotoxic chemotherapy (maximum tolerated dose, MTD) which is rarely reconsidered after approval or labelling.^32^ Modern targeted therapies bind to specific molecules, and often there is no increase in efficacy beyond a certain dose, making the MTD concept irrelevant for new generation of anticancer agents.^32^ Phase I trials of enzalutamide, apalutamide and darolutamide do not show evidence of a relationship between increased drug exposure and efficacy near the labelled dose, suggesting that lower doses of these drugs may have near-equivalent efficacy in metastatic prostate cancer.^3 7 33^

Cancer care is challenged by high drug expenditures and an ageing population,^32^ and it is important to identify safe, effective, less expensive dosing regimens.^32 34^ There is substantial financial toxicity associated with the ’utamides to patients (where medication costs are not covered) and to healthcare payers. Daily doses of enzalutamide, apalutamide and darolutamide are given with multiple pills, so reducing the dose may be a simple strategy, and if shown equally effective, may increase access to treatment and reduce financial toxicity.^34^ Dose reduction to improve tolerability or to counteract potential drug–drug interaction with existing medication may derive additional health economic benefit from cost-savings and improvement in quality of life from fewer, less severe toxic effects.

In this review, we have summarised preliminary evidence to suggest that lower doses of the ’utamides can maintain efficacy and reduce toxicity. There are also several meeting reports^12 35 36^ and individual case reports^37^,^39^ that show good or maintained oncological responses with low doses of enzalutamide. Other limited retrospective studies of anti-androgen drugs found that lower doses did not decrease the incidence of adverse events but did result in lower PSA response rates, although this did not impact on clinical disease progression or OS.^26 40 41^

Prostate cancer is common in older men, who are vulnerable to side-effects and drug interactions with existing medications. A few small studies have explored the feasibility of using reduced-dose enzalutamide, apalutamide or darolutamide in elderly patients and those with poor ECOG performance status,^35 36 42^ and report comparable survival outcomes and fewer treatment-related adverse events. However, sample numbers in these studies were small, the duration of follow-up limited and the studies were potentially subject to confounding by indication; the results need to be validated in a prospective study using prespecified reduced anti-androgen drug doses. Alternative approaches to de-escalation in the treatment of metastatic castrate-sensitive prostate cancer to balance both the benefits and long-term risks and burden of treatment are under exploration.^43 44^

A limitation of this rapid review is that the results may be susceptible to selection bias, systematic error in the assessment and synthesis of results, or the omission of key studies.^19^ The search strategy was limited to English-language and full-text studies only, and it did not include a formal quality assessment of the risk of bias, due to the rapid nature of the review, the range of studies included and limited tools available for appraising phase I studies.

Standard dosing of enzalutamide, apalutamide and darolutamide may require reduction because of adverse events, but there are limited data on long-term clinical outcomes at lower doses. This review provides evidence that enzalutamide, apalutamide or darolutamide could be given at a lower than the standard recommended dose without likely loss of antitumour activity, and lower doses may lead to a decrease in toxicity. A prospective randomised trial comparing reduced and standard doses of enzalutamide, apalutamide and darolutamide is needed to investigate the efficacy of lower-dose regimens.

## Supplementary material

10.1136/bmjonc-2023-000198 online supplemental file 1

## Data Availability

Data sharing not applicable as no datasets generated and/or analysed for this study. All data relevant to the study are included in the article or uploaded as supplemental information.
