# Supplementary material for: Comparison of standard-dose and reduced-dose treatment of metastatic prostate cancer with enzalutamide, apalutamide or darolutamide: a rapid review
Source: BMJ Oncol. 2024 Feb 29;3(1):e000198. doi: 10.1136/bmjonc-2023-000198 (PMC11235015; doi:10.1136/bmjonc-2023-000198)
Supplement: online supplemental file 1 [file bmjonc-2023-000198supp001.pdf]

<u>Supplemental material (S1)</u>

S1: Search strategy used in the rapid review

Database: Embase <1974 to 2023 June 18>, Ovid MEDLINE(R) ALL <1946 to June 18, 2023>
Search Strategy:

1 (prostat* adj3 (cancer* or tumo* or neoplas* or carcinom* or malign*)).tw.

2 Prostatic Neoplasms/ or prostate cancer/

3 (enzalutamide or apalutamide or darolutamide or Xtandi or Erleada or Nubeqa).mp.

4 Androgen Antagonists/ or Androgen Receptor Antagonists/ or enzalutamide/ or apalutamide/ or darolutamide/ or androgen receptor antagonist/ or antiandrogen/ (37758)

5 ((dose or doses or dosage* or medication) adj1 (change* or changing or reduce* or reduction or escalation or response)).tw. or "Dose-Response Relationship, Drug"/ or drug dose regimen/ or drug dose reduction/ or drug underdose/ or recommended drug dose/

6 1 OR 2

7 3 OR 4

8 5 AND 6 AND 7

9 Remove duplicates from 8

S2: Detailed summary of included studies (n=10)

Study	Drug and Dose	Population	Methods and design	Adverse events	Key Outcomes
Akaza (Akaza et al., 2016) 2016<br><br>Phase I/II trial	Enzalutamide<br><br>Phase I: 80mg, 160mg, 240mg/day<br><br>Phase II: 160mg/day	Patients with metastatic castrate resistant prostate cancer, post doxetaxel<br><br>ECOG 0-1	• Multi-centre open-label uncontrolled phase I/II trial in Japan<br>• In phase I, 9 patients enrolled into dose-escalation study, (three patients assigned to 80, 160 or 240 mg/day groups).<br>• In phase II, 38 patients assigned to 160mg/day (dose expansion).	AE related to drug:<br>• Phase I 7 (78%): anorexia, constipation, hypertension, nausea, fatigue, pyrexia<br>• Phase II 31 (66%): weight loss, anorexia, constipation, hypertension, pain, nausea, fatigue, rash, prolonged QTc	• During phase I, enzalutamide was well tolerated in each cohort; median duration of exposure in each group was 584.0, 171.0 and 252.0 days.<br>• Pharmacokinetics were dose-proportional after a single dose ranging from 80 to 240mg.<br>• Prostate-specific antigen response rate (≥50 % reduction from baseline) was 28.9% at 160mg/day at 12 weeks.<br>• Fewer serious treatment-emergent adverse events reported in phase I at lower doses (22.2% vs 34.2%).
Fizazi (Fizazi et al., 2014) 2014<br><br>Phase I/II trial	Daroluaimide<br><br>Phase 1: starting daily dose of 200mg, increased to 200-1800mg<br><br>Phase 2: 200 mg, 400 mg, and 1400 mg	Men with progressive metastatic castration-resistant prostate cancer (CRPC)<br><br>ECOG 0-1	• ARCADES trial: open label multi-centre Phase I (dose escalation)/ Phase II (dose expansion) trial in 23 hospitals across Europe and USA<br>• In phase I, 24 patients were enrolled to six sequential cohorts of three to six patients, and received a starting daily dose of 200mg, increased to 400mg, 600mg, 1000mg, 1400mg and 1800mg.<br>• In phase II, 110 patients randomly assigned to three groups: 200 mg (n=38), 400 mg (n=37), and 1400 mg (n=35).	• In phase I, three patients reported eight adverse events of grade 3 and one patient had a grade 4 adverse event (lymphoedema), none of which were deemed to be related to treatment.<br>• Phase II: most common AE were	• Primary endpoint safety and tolerability (Phase I) and proportion with a PSA response (50% or greater decrease in serum PSA) at week 12 in Phase II.<br>• In phase 1, no dose-limiting toxic effects were reported and the maximum tolerated dose was not reached.<br>• In phase II, 38 patients who received 200 mg, 39 who received 400 mg, and 33 who received 1400 mg were assessable for PSA response at 12 weeks.

			• Of the phase I patients, four who received 200 mg, seven who received 400 mg, and three who received 1400 mg also entered the phase II trial, resulting in total N = 124.	fatigue (12%) or hot flushes (5%).<br>• One severe (grade 3 fatigue) event related to the drug.<br>• No differences observed when examined by dose.	• Phase II: 11 (29%) patients in the 200 mg group, 13 (33%) in the 400 mg group, and 11 (33%) in the 1400 mg group achieved a PSA response at 12 weeks.
Fizazi (Fizazi et al., 2017) 2017<br><br>Phase I/II trial (extended follow up)	Darolutamide<br><br>200-1800mg	Patients with castration-resistant prostate cancer (CRPC).<br><br>Extended follow-up in CYP17 inhibitor (CYP17i)-naive patients.	• Extended follow up in CYP17 inhibitor naïve patients from ARCADES trial.<br>• Multicentre phase I/II trial of 134 patients, 77 of who were deemed CYP17 inhibitor naïve.<br>• Patients (n = 77) received oral ODM-201 twice daily at daily doses of 200-1800 mg	• The majority of AEs (61.1%) were mild (grade 1); the most common AE was fatigue/asthenia (35.1% of patients), with no clear relationship to darolutamide.<br>• ≥1 adverse event in 75/77 (97.4%) patients.<br>• Treatment related AEs occurred in 27 patients (35.1%)<br>• Commonest treatment related AE were fatigue (10.4%), diarrhoea (5,2%), anorexia (5.2%) and hot flushes (3.9%)	• Median treatment duration 8.2 months (95% CI 5.6-11.0).<br>• Median time to PSA progression was 25.2 months (95% CI 11.3-25.2) for chemotherapy-naive men and not reached (NR; 95% CI 5.5-NR) for chemotherapy-pretreated patients; a trend for improved antitumour response was observed for chemotherapy-naive patients.<br>• Median time to radiographic progression was longer for chemotherapy-naive (14.0 mo, 95% CI 8.1-33.3) than for chemotherapy-pretreated (7.2 mo, 95% CI 2.7-11.0) patients.<br>• For patients receiving expanded dose levels (n = 68; 200, 400 and 1400 mg/d), the HR for PSA progression was 0.47 (95% CI 0.12–1.82; p = 0.2743) for the 1400 mg/d group and 1.32 (95% CI 0.48–

					3.62; p = 0.596) for the 400 mg/d group when compared with the 200 mg/d group.
Freedland (Freedland et al., 2021) 2021<br><br>Retrospective cohort study	Enzalutamide<br><br>Reduced dose enzalutamide (<80%)	Patients with metastatic castration-resistant prostate cancer (mCRPC).	• Longitudinal cohort study of 6069 US Veteran health electronic data between 2010 and 2016.<br>• PSA progression defined as first rise in PSA >2 ng/mL and 25% above nadir.<br>• Relative dose intensity (RDI) defined as ratio of total dispensed dose over the last two months to the standard recommended dose.<br>• Dose reduction assessed using a threshold of RDI <80%.	• No data on adverse events was reported in the main analysis	• Mean follow up 12.3 months<br>• 67.2% enzalutamide patients had >1 occurrence of RDI <80%, with averaged dose reduction of enzalutamide at 59% standard 160mg/day dose.<br>• Multivariate Cox proportional hazards model suggested an RDI <80% was associated with statistically significant higher risk of PSA progression HR [95% CI 1.258 [1.080–1.464], p<0.003)<br>• No assessment of PFS or OS reported
Oishi (Oishi et al., 2022) 2022<br><br>Retrospective analysis	Apalutamide<br><br>240mg or reduced dose (60-180mg/day)	35 patients with non-metastatic CRPC and 72 patients with treatment-naive metastatic castration-sensitive prostate cancer	• Multi-centre retrospective study of 107 patients (35nmCRPC and 72 mCRPC) treated with apalutamide between June 2019-2022.<br>• Classified into two groups: those treated with standard dose (240mg/day) n = 65 (67%), and those treated with reduced dose (60-180mg/day) n = 42 (39.3%).<br>• Median apalutamide dose in reduced group 180mg/day.<br>• Primary outcome was the effect of apalutamide dose reduction on skin related adverse events.	• Incidence of skin-related AEs in the full dose (55.4%; n = 36) and reduced dose (42.9%; n = 18) groups was not significantly different (p = 0.761)<br>• Treatment-related AEs other than skin related AEs were observed in 6 (5.6%) patients: fatigue, hepatic impairment, palpitation, interstitial	• Overall skin-related AE rate was not significantly different between dose groups (55% vs. 43%, p=0.761).<br>• But incidence of skin-related AEs was significantly lower in patients with small body sizes (body weight <67kg and body mass index <24kg/m2) in the reduced dose apalutamide group (p 0.032).<br>• In the 72 patients with mCSPC, CRPC-free survival was not significantly different between the full and reduced dose apalutmaide groups.<br>• Skin-related AE-free survival was not significantly different between standard

				pneumonia, and renal dysfunction.	and reduced dose apalutamide groups (p = 0.33)<br>• Apalutamide discontinuation was significantly higher in patients receiving the standard dose, n = 18 (50%), than the reduced dose n -3, (16.7%) group.
Rathkopf (Rathkopf et al., 2013) 2013<br><br>Phase I study	Apalutamide<br><br>Patients were assigned sequentially to escalating dose levels of apalutamide, between 30-480mg	Patients with progressive metastatic CRPC	• Phase I study of 30 patients with progressive CRPC received continuous daily oral apalutamide at doses between 30 and 480mg.<br>• Patients were assigned to nine escalating dose levels (30, 60, 90, 120, 180, 240, 300, 390 and 480 mg/day)<br>• PET computed tomography imaging was conducted to monitor FDHT binding to androgen receptors in tumours before and during treatment.<br>• Primary objective to determine pharmacokinetics, safety and recommended phase II dose.	• The most frequently reported adverse event was grade 1/2 fatigue (47%).<br>• Other AEs: pain, diarrhoea, nausea, constipation, hot flushes, dyspnoea, peripheral neuropathy, peripheral oedema<br>• One dose-limiting toxicity event (grade 3 abdominal pain) at 300mg dose.	• Pharmacokinetics were linear and dose proportional.<br>• PSA decline at 12 weeks (≥50% reduction from baseline) observed in 46.7% patients.<br>• Reduction in FDHT uptake observed at all doses, with a plateau in response at ≥120-mg dose, consistent with saturation of AR binding.<br>• Dose escalation to 480 mg did not identify a maximum-tolerated dose.
Scher (Scher et al., 2010) 2010<br><br>Phase I/II study	Enzalutamide<br><br>Dose escalation from 30-600mg	Patients with progressive, metastatic castration-resistant prostate cancer	• Phase I/II study undertaken in 140 patients in USA.<br>• Dose-escalation cohorts of three to six patients, starting at 30mg/day.<br>• Final doses studies were: 30 mg (n=3), 60 mg (27), 150 mg (28), 240 mg (29), 360 mg (28), 480 mg (22), and 600 mg (3)	• The most common grade 3–4 adverse event was dose-dependent fatigue (16 [11%] patients), which generally resolved after dose reduction.	• Anti-tumour effects were observed at all doses, including decreases in serum PSA of ≥50% in 78 (56%) patients, responses in soft tissue in 13 (22%) of 59 patients, stabilised bone disease in 61 (56%) of 109 patients, and conversion from unfavourable to favourable circulating tumour cell counts in 25 (49%) patients.

			• Primary objective was to identify the safety and tolerability of enzalutamide and to establish the maximum tolerated dose.	• Fatigue highest in doses ≥240mg/day<br>• Commonest overall AEs: fatigue, nausea, dyspnoea, anorexia and back pain.<br>• Two seizures occurred in patients receiving doses of 600mg and 360mg/day.	• PET imaging of 22 patients to assess androgen-receptor blockade showed decreased binding at doses from 60 mg to 480 mg per day (range 20–100%).<br>• Median time to progression was 47 weeks (95% CI 34–not reached) for radiological progression.<br>• Maximum tolerated dose for sustained treatment (>28 days) was 240mg.
T'Jollyn (T'Jollyn et al., 2022) 2022<br><br>Retrospective Phase III analysis	Apalutamide<br><br>Standard dose 240mg/day<br><br>Compared with quartiles of dose exposure based	Patients with metastatic castration-sensitive prostate cancer<br><br>ECOG 0-1	• Retrospective analysis of 1052 patients in phase III TITAN trial<br>• 1052 patients were randomised to Apalutamide + androgen-deprivation therapy (n=525) or Placebo + androgen-deprivation therapy (n=527).<br>• Cox regression analysis investigated the relationships between apalutamide exposure and overall survival and exposure and overall survival and radiographic progression-free survival at apalutamide exposure quartiles.	• Incidence of skin rash and pruritis increased significantly with increasing apalutamide exposure.	• No statistical association was detected between overall survival and apalutamide exposure quartiles.<br>• No statistical association was detected between radiographic progression-free survival and apalutamide exposure quartiles, within a narrow apalutamide exposure range (coefficient of variation 22%).
Vinh-Hung (Vinh-Hung et al., 2022) 2022<br><br>Retrospective analysis	Enzalutamide<br><br>Standard dose (160mg/day) vs low-dose (≤80mg/day)	Patients with metastatic prostate cancer	• Retrospective review of 111 patients with metastatic prostate cancer treated with enzalutamide at a Caribbean single centre in 2014-2020.<br>• 79 patients received standard dose (160mg/day) and 32 patients received low-dose (≤80mg/day) enzalutamide.	• Limited data reported in the analysis	• Patients taking low-dose enzalutamide reported less prior chemotherapy exposure, worse ECOG performance status, more comorbidities, including cardiovascular disease.<br>• OS and PFS did not differ between low-dose and standard dose groups.

			• Primary outcome restricted mean survival time and restricted mean attained age using Irwin methods<br>• Secondary outcomes were OS, PFS, PSA progression.		• Patients on low-dose enzalutamide had a better longevity with significantly longer RMAA, 89.1 years, versus standard-dose RMAA of 83.8 years (P = .003).<br>• In a subgroup analysis by age at start of enzalutamide, < 75 versus ≥75 years, longevity was also better with low-dose in younger patients (Δ = 2.9 years, P = .034, and older, Δ = 3.3 years, P = .011)
Vinh-Hung (Vinh-Hung et al., 2020) 2020<br><br>Retrospective analysis	Enzalutamide<br><br>Standard dose (160mg/day) vs low-dose (≤80mg/day)	Patients aged ≥75 years with metastatic prostate cancer	• Retrospective review of 59 patients with metastatic prostate cancer treated with enzalutamide at a single centre in Caribbean between 2014-2020.<br>• Of 59 patients, 43 received standard dose (160mg/day) and 16 patients received low-dose (≤80mg/day) enzalutamide.<br>• Median follow up of 1 year.	• No adverse cardiovascular or neurological events were reported in the low dose group.<br>• Grade 4 fatigue reported in two patients in the standard dose group.	• PSA decrease of ≥50% at 12 weeks was observed in 67% patients (10/15), versus 45% with standard dose.<br>• Median progression-free survival was 11.2 months in low-dose group, versus 11.9 months for patients receiving the standard dose (P = 0.612).

Supplementary material 3: Further PK/PD data from phase I studies

In phase I/II studies, the pharmacokinetic profiles of enzalutamide were linear by dose range (Scher et al. 2010). Half-life was approximately 1 week and not affected by dose. Androgen-receptor binding was observed at all doses. Despite an almost linear increase in serum concentration with dose, no significant increase to target binding was observed from 60mg to 360mg per day on FDHT-PET scan. PSA decrease from baseline at 12 weeks were observed at all doses; the extent of decrease and proportion of patients recording a fall in PSA was dependent on the dose up to 150mg/day, but there was no clear additional benefit recorded for higher doses.

Pharmacokinetics (peak plasma concentrations and AUC) of apalutamide were reported as dose proportional in phase I/II studies (Rathkopf et al. 2013). Plasma concentrations declined slowly, with a mean half-life at steady-state of 3-4 days. Drug half-life and time to steady-state were independent of dose. Plasma trough concentrations increased steadily with time in proportion to dose, with most patients reaching steady-state exposure following 3 weeks continuous apalutamide. Reduction in FDHT-PET/CT uptake (used to measure pharmacodynamic response) was observed at all doses, with a plateau in response at 120mg/day, consistent with saturation of AR binding. At 12 weeks, 14 (47%) patients had >50% decline in PSA compared with baseline.

In phase I studies, exposure of darolutamide at steady state increased in a linear, dose-related pattern up to 1400mg/day with steady state plasma concentration reached after 1 week of continuous treatment (Fizazi et al. 2014). Anticancer activity was noted across all doses. A PSA response (≥50% decrease in PSA) was observed by week 12 at all doses, although responses were lower in patients previously treated with CYP17 inhibitors and in patients who had previously received other chemotherapy. Median time to PSA progression was not reached in phase I-II trials at 12 weeks at all dose levels. No clear differences in in radiological soft tissue and bone disease response were noted by darolutamide dose at 12 weeks; median time to radiological progression was not reached for patients who were naïve to any chemotherapy (including CYP17 inhibitor). No clear differences were observed in response to dose darolutamide and circulating tumour cell levels at 12 weeks. Compared with baseline, 41 (82%) patients maintained favourable circulating tumour cell counts (<5 cells per 7.5ml of blood), although no clear differentiation by dose was defined. Extended follow up of the sub-group of 77 patients with CYP17 inhibitor naïve prostate cancer reported anti-cancer activity at all doses (Fizazi et al. 2017). The median time to PSA progression was not reached for in the sub-group of chemotherapy naïve patients receiving 200mg and 1400 mg/day of darolutamide, and was reported as 25.2 months (95% CI 4.7–25.2, IQR 4.7-25.2) for patients receiving 400 mg/day.

Supplementary Material References

AKAZA, H., UEMURA, H., TSUKAMOTO, T., OZONO, S., OGAWA, O., SAKAI, H., OYA, M., NAMIKI, M., FUKASAWA, S., YAMAGUCHI, A., UEMURA, H., OHASHI, Y., MAEDA, H., SAITO, A., TAKEDA, K. & NAITO, S. 2016. A multicenter phase I/II study of enzalutamide in Japanese patients with castration-resistant prostate cancer. *Int J Clin Oncol,* 21**,** 773-782.

FIZAZI, K., MASSARD, C., BONO, P., JONES, R., KATAJA, V., JAMES, N., GARCIA, J. A., PROTHEROE, A., TAMMELA, T. L., ELLIOTT, T., MATTILA, L., ASPEGREN, J., VUORELA, A., LANGMUIR, P. & MUSTONEN, M. 2014. Activity and safety of ODM-201 in patients with progressive metastatic castration-resistant prostate cancer (ARADES): An open-label phase 1 dose-escalation and randomised phase 2 dose expansion trial. *The Lancet Oncology,* 15**,** 975-985.

FIZAZI, K., MASSARD, C., BONO, P., KATAJA, V., JAMES, N., TAMMELA, T. L., JOENSUU, H., ASPEGREN, J. & MUSTONEN, M. 2017. Safety and Antitumour Activity of ODM-201 (BAY-1841788) in Castration-resistant, CYP17 Inhibitor-naive Prostate Cancer: Results from Extended Follow-up of the ARADES Trial. *European Urology Focus,* 3**,** 606-614.

FREEDLAND, S. J., LI, S., PILON, D., BHAK, R. H., NARKHEDE, S., LEFEBVRE, P. & YOUNG-XU, Y. 2021. Medication patterns of abiraterone acetate plus prednisone or enzalutamide and PSA progression in veterans with metastatic castration-resistant prostate cancer. *Current Medical Research and Opinion,* 37**,** 635-642.

OISHI, T., HATAKEYAMA, S., TABATA, R., FUJIMORI, D., KAWASHIMA, Y., TANAKA, R., ISHII, N., MIURA, H., TANAKA, T., OKAMOTO, T., YAMAMOTO, H., YONEYAMA, T., HASHIMOTO, Y., SATO, S. & OHYAMA, C. 2022. Effects of apalutamide dose reduction on skin-related adverse events in patients with advanced prostate cancer: A multicenter retrospective study. *The Prostate*.

RATHKOPF, D. E., MORRIS, M. J., FOX, J. J., DANILA, D. C., SLOVIN, S. F., HAGER, J. H., RIX, P. J., CHOW MANEVAL, E., CHEN, I., GONEN, M., FLEISHER, M., LARSON, S. M., SAWYERS, C. L. & SCHER, H. I. 2013. Phase I study of ARN-509, a novel antiandrogen, in the treatment of castration-resistant prostate cancer. *Journal of clinical oncology : official journal of the American Society of Clinical Oncology,* 31**,** 3525-3530.

SCHER, H. I., ANAND, A., RATHKOPF, D., SHELKEY, J., MORRIS, M. J., DANILA, D. C., LARSON, S., HUMM, J., FLEISHER, M., SAWYERS, C. L., BEER, T. M., ALUMKAL, J., HIGANO, C. S., YU, E. Y., TAPLIN, M. E., EFSTATHIOU, E., HUNG, D., HIRMAND, M. & SEELY, L. 2010. Antitumour activity of MDV3100 in castration-resistant prostate cancer: A phase 1-2 study. *The Lancet,* 375**,** 1437-1446.

T'JOLLYN, H., ACKAERT, O., CHIEN, C., LOPEZ-GITLITZ, A., MCCARTHY, S., RUIXO, C. P., KARSH, L., CHI, K., CHOWDHURY, S., RUIXO, J. J. P. & AGARWAL, N. 2022. Efficacy and safety exposure-response relationships of apalutamide in patients with metastatic castration-sensitive prostate cancer: results from the phase 3 TITAN study. *Cancer Chemotherapy and Pharmacology,* 89**,** 629-641.

VINH-HUNG, V., GOROBETS, O., NATCHAGANDE, G., SARGOS, P., YIN, M., NGUYEN, N. P., VERSCHRAEGEN, C. & FOLEFAC, E. 2022. Low-Dose Enzalutamide in Metastatic Prostate Cancer-Longevity Over Conventional Survival Analysis. *Clinical Genitourinary Cancer,* 20**,** e473-e484.

VINH-HUNG, V., NATCHAGANDE, G., JOACHIM, C., GOROBETS, O., DRAME, M., BOUGAS, S., FOLEFAC, E., NGUYEN, N. P., VERSCHRAEGEN, C. & YIN, M. 2020. Low-Dose Enzalutamide in Late-Elderly Patients (>= 75 Years Old) Presenting With Metastatic Castration-Resistant Prostate Cancer. *Clinical Genitourinary Cancer,* 18**,** e660-e668.
